# Global small-angle scattering data analysis of inverted hexagonal phases

**DOI:** 10.1107/S1600576719002760

**Published:** 2019-03-28

**Authors:** Moritz P. K. Frewein, Michael Rumetshofer, Georg Pabst

**Affiliations:** a University of Graz, Institute of Molecular Biosciences, Biophysics Division, NAWI Graz, 8010 Graz, Austria; b BioTechMed Graz, 8010 Graz, Austria; c Graz University of Technology, Institute of Theoretical Physics and Computational Physics, NAWI Graz, 8010 Graz, Austria

**Keywords:** full-*q*-range analysis, inverted hexagonal phases, phospholipids, intrinsic curvature, Bayesian analysis

## Abstract

A global analysis model has been developed for randomly oriented, fully hydrated, inverted hexagonal (H_II_) phases formed by many amphiphiles in aqueous solution, including membrane lipids. The model is based on a structure factor for hexagonally packed rods and a compositional model for the scattering length density, enabling also the analysis of positionally weakly correlated H_II_ phases. Bayesian probability theory was used for optimization of the adjustable parameters, which allows parameter correlations to be retrieved in much more detail than standard analysis techniques and thereby enables a realistic error analysis. The model was applied to different phosphatidylethanolamines, including previously unreported H_II_ data for diC14:0 and diC16:1 phosphatid­yl­ethanolamine. The extracted structural features include intrinsic lipid curvature, hydrocarbon chain length and area per lipid at the position of the neutral plane.

## Abstract 2

A global small-angle scattering model for unoriented, fully hydrated, inverted hexagonal phases is provided. The model is evaluated using Bayesian probability theory to obtain reliable estimates for the structural parameters.

## 1. Introduction   

Elastic small-angle scattering (SAS) techniques are unrivaled for providing detailed structural insight into aggregates formed by amphiphiles in aqueous solutions (Glatter, 2018 ▸). In the field of membrane biophysics, significant efforts have been devoted to the development of SAS analysis methods for the biologically most relevant fluid lamellar phases, including domain-forming lipid mixtures and asymmetric lipid bilayers (Heberle & Pabst, 2017 ▸). In contrast, non-lamellar phases such as the inverted hexagonal (H_II_) phase are less commonly found for membrane lipids under physiological conditions but are of significant biotechnological interest, for example, for gene transfection (Koltover *et al.*, 1998 ▸) or drug delivery systems (Yaghmur & Glatter, 2009 ▸). H_II_ phases are also highly amenable systems for deriving intrinsic lipid curvatures by small-angle X-ray scattering (SAXS) (Leikin *et al.*, 1996 ▸; Di Gregorio & Mariani, 2005 ▸; Kollmitzer *et al.*, 2013 ▸; Chen *et al.*, 2015 ▸), which is the main focus of the present contribution. The intrinsic lipid curvature *C*_0_ is given by the negative inverse of the curvature radius, −1/*R*_0_, of an unstressed monolayer at the position of the neutral plane, which corresponds to the location where molecular bending and stretching modes are decoupled (Kozlov & Winterhalter, 1991 ▸). Major interest in obtaining reliable *C*_0_ values originates from its contribution to the stored elastic energy strain in planar bilayers (Marsh, 2006 ▸), transmembrane protein function (Dan & Safran, 1998 ▸; Frewein *et al.*, 2016 ▸) and overall membrane shape (Frolov *et al.*, 2011 ▸).

Structural details of H_II_ phases have been successfully derived using electron density map reconstruction based on Bragg peak scattering only (Tate & Gruner, 1989 ▸, 1992 ▸; Rand *et al.*, 1990 ▸; Harper *et al.*, 2001 ▸). However, for highly swollen H_II_ phases or at elevated temperatures the number of observed Bragg peaks may become insufficient for a reliable analysis. This may be particularly the case for mixtures of cone-shaped (H_II_-phase-forming) and cylindrically shaped (lamellar-phase-forming) or inverted cone-shaped (spherical-micelle-forming) lipids. Such mixtures are typically used for determining *C*_0_ for non-H_II_-phase-forming lipids [see *e.g.* Kollmitzer *et al.* (2013 ▸)]. In this case global analysis techniques which take into account both Bragg peaks and diffuse scattering become advantageous, as demonstrated previously also for lamellar phases (Pabst *et al.*, 2000 ▸).

Global analysis techniques have been reported previously for H_I_ phases, *i.e.* oil-in-water-type hexagonal aggregates (Freiberger & Glatter, 2006 ▸; Sundblom *et al.*, 2009 ▸). The specific need for developing a dedicated model for H_II_ phases comes from the observation that unoriented H_II_ phases contain previously unreported additional diffuse scattering originating most probably from packing defects. We have evaluated our global H_II_ model for phosphatidylethanol­amines with differing hydrocarbon chain composition and as a function of temperature using Bayesian probability theory to increase the robustness of analysis. This method significantly increased the obtained information content compared with our previous analysis (Kollmitzer *et al.*, 2013 ▸) and allowed us to derive details about the structure, for example the lipid headgroup area, hydrocarbon chain length and molecular shape to name but a few.

## 2. Experimental methods   

### 2.1. Sample preparation   

Dioleoyl phosphatidylethanolamine (DOPE, diC18:1 PE), palmitoyl oleoyl phosphatidylethanolamine (POPE, C16:0-18:1 PE), dimyristoyl phosphatidylethanolamine (DMPE, diC14:0 PE) and dipalmitoleoyl phosphatidylethanolamine (diC16:1 PE) were purchased in the form of powder from Avanti Polar Lipids (Alabaster, AL, USA). *cis*-9-Tricosene was obtained from Sigma–Aldrich (Vienna, Austria). All lipids were used without any further purification. Note that dipalmitoleoyl phosphatidylethanolamine is deliberately abbreviated to diC16:1 PE in order not to be confused with dipalmitoyl phosphatidylethanolamine (diC16:0 PE).

Fully hydrated H_II_ phases were prepared using rapid solvent exchange (RSE) (Buboltz & Feigenson, 1999 ▸) as detailed previously (Leber *et al.*, 2018 ▸). In brief, stock solutions of lipids (10 mg ml^−1^) and tricosene (5 mg ml^−1^) were first prepared by dissolving both compounds in chloroform/methanol (9:1 *v*/*v*). Ultra-pure water (18 MΩ cm^−2^) was filled into test tubes and equilibrated at 333–342 K using an incubator. Lipid and tricosene stock solutions were added to the test tubes containing preheated water (organic solvent/water ratio = 2.55) and then quickly mounted onto the RSE apparatus, described by Rieder *et al.* (2015 ▸). The organic solvent quickly evaporated under the following settings: temperature: 338 K; vortex speed: 600 r min^−1^; argon flow: 60 ml min^−1^; and final vacuum pressure: 400–500 mbar (1 mbar = 100 Pa). The full procedure was performed for 5 min, yielding a lipid pellet at the bottom of the test tube in excess water. All samples contained 12 wt% tricosene. Tricosene inserts preferentially into the interstical space between the rods in H_II_ phases, effectively reducing packing frustration as verified previously (Alley *et al.*, 2008 ▸; Kollmitzer *et al.*, 2013 ▸). Unstressed H_II_ phases are required for *C*_0_ determination (Kozlov & Winterhalter, 1991 ▸).

### 2.2. Scattering experiments   

Small-angle X-ray scattering (SAXS) experiments were performed via a SAXSpace compact camera (Anton Paar, Graz, Austria) equipped with an Eiger R 1 M detector system (Dectris, Baden-Daettwil, Switzerland) and a 30 W-Genix 3D microfocus X-ray generator (Xenocs, Sassenage, France), supplying Cu *K*α (λ = 1.54 Å) radiation with a circular spot size of the beam of ∼300 µm on the detector. Samples were taken up in paste cells (Anton Paar) and equilibrated at each measured temperature for 10 min using a Peltier controlled sample stage (TC 150, Anton Paar). The total exposure time was 32 min (four frames of 8 min), with the sample-to-detector distance set to 308 mm. Data reduction, including sectorial data integration and corrections for sample transmission and background scattering, was performed using the program *SAXSanalyis* (Anton Paar).

## 3. Model   

### 3.1. General aspects for H_II_ phases   

We initially tested the applicability of a previously reported model-free approach (Freiberger & Glatter, 2006 ▸). However, although perfect fits to the experimental data were obtained, the corresponding pair distance distribution functions contained significantly negative values upon approaching the maximum particle size (*D*_max_), which is not physically relevant (O. Glatter, personal communication). This encouraged us to proceed with data modeling.

**Figure 1:**
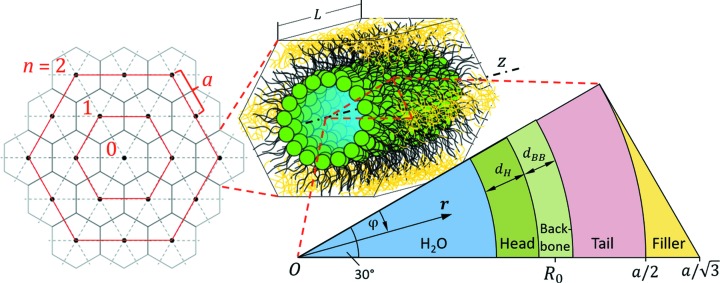
Scheme of the H_II_ phase model. The hexagonal lattice (left side) is defined by its lattice parameter *a* and the number of rings (lattice order) *n*. Its unit cell, shown in the center, is a regular hexagonal prism of length *L* and consists of a cylindrical water core, surrounded by lipids with their heads pointing toward the central water channel and a filler molecule occupying the interstices. We denote the axis of rotation by *z*. The unit cell is subdivided into areas of different SLD which depend on the molecular composition (see also Fig. 3). *R*_0_ denotes the position of the neutral plane at the center of the lipid backbone.

To do so, we considered a bundle of hexagonal prisms consisting of a water core coated by lipids (Fig. 1 ▸). Its scattering intensity is characterized by the form factor of a single prism *F*(**q**) and the structure factor of the whole bundle *S*(**q**), where **q** is the scattering vector. Assuming that the prisms are long as compared to their diameters allows us to decouple form and structure factors (Freiberger & Glatter, 2006 ▸): 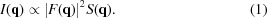



The structure factor of a two-dimensional lattice of infinitely long hexagonal prisms, averaged over all in-plane vectors, is given by (Oster & Riley, 1952 ▸; Marchal & Demé, 2003 ▸; Freiberger & Glatter, 2006 ▸) 
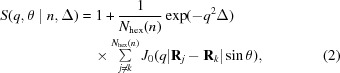
where θ is the angle between the scattering vector and the axis (*z*) normal to the hexagons, *N*_hex_ = 1 + 3*n*(*n* + 1) is the total number of unit cells for *n* rings (Fig. 1 ▸), and *J*_0_ is the zero-order Bessel function of the first kind. The exp(−*q* ^2^Δ) term is well known as the Debye–Waller factor, where Δ is the lateral mean-square displacement of the rotation axes of the unit cells around their mean positions **R**_*j*_. For the sake of readability we give the parameter dependencies after the vertical line in each equation, *i.e.* for equation (2) *n* and Δ. Analogously to Freiberger & Glatter (2006 ▸) we also considered a polydispersity of 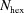, which yields a smooth structure factor. However, since this affects only low *q* values and not significantly the final quality of the result, this was omitted in order to reduce overall computation times. An alternative structure factor, based on the positioning of peaks with flexible shapes on hexagonal lattice points, has been reported previously (Förster *et al.*, 2005 ▸; Sundblom *et al.*, 2009 ▸). However, its application involves a significantly higher number of adjustable parameters, which leads us to disregard this option.

The form factor for a hexagonal prism of length *L* is given by (Freiberger & Glatter, 2006 ▸) 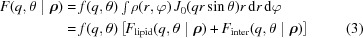
with 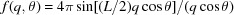, where ρ(*r*, φ) is the in-plane scattering length density (SLD). Here, 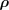 denotes all parameters describing the SLD ρ(*r*, φ). For evaluation, which because of symmetry is performed over 1/12 of the area of the hexagon, the form factor is split into a core–shell cylindrical part 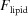, which accounts for the phospholipid only, and 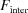, which accounts for the interstitial space, often taken up by pure hydrophobic filler molecules (here, tricosene). We also found that the length of the cylinders does not affect *F* significantly for *L* ≥ 2500 Å for cylinder radii between 35 and 45 Å, as occurring in the present paper. To shorten the computational times, we therefore fixed *L* = 2500 Å for all our further calculations.

The core–shell cylindrical part can be evaluated analytically (Székely *et al.*, 2010 ▸): 
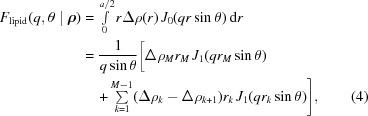
where *M* is the total number of shells, *r*_*k*_ are the shell radii, Δρ is the SLD relative to water (Δρ = ρ − ρ_W_; ρ_W_ = 0.33 Å^−3^ in the case of X-rays) and *J*_1_ is the first-order Bessel function of the first kind.

Molecular fluctuations cause a smearing of the sharp boundaries between the individual slabs. Analogously to the approach adopted by Franks *et al.* (1982 ▸), these were taken into account by translating all shell boundaries {*r*_*k*_} by the distance *x*, whose value was assumed to be distributed by a Gaussian 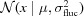 of mean μ and variance 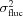:
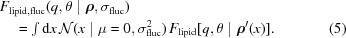
Here, μ = 0 and 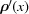 denotes the SLD including the radial shift *x*.

The form factor of the interstices 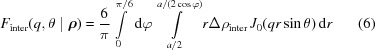
needs to be evaluated numerically, but remains constant for a given lattice constant *a* and SLD 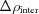. However, since *a* can be determined accurately from Bragg peak positions, 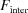 needs to be calculated only once for each scattering pattern.

H_II_ phases are well known to change their phase from ‘+’ to ‘−’ between the 10 and 11 reflections (Turner & Gruner, 1992 ▸), which brings about a minimum in 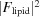 between the two peaks.

**Figure 2:**
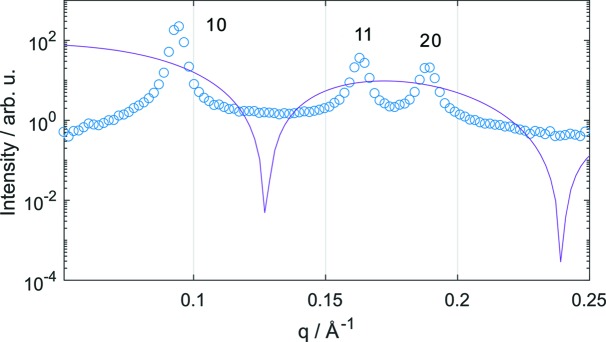
Overlay of the scattering pattern of DOPE (circles) and 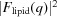 (solid line). The phase change between the 10 and 11 reflections for the H_II_ phase leads to a minimum in the absolute square of the form factor, which is absent in the experimental data.

All our present experimental data, as well as those previously reported (Kollmitzer *et al.*, 2013 ▸; Leber *et al.*, 2018 ▸), exhibited significant diffuse scattering between reflections 10 and 11 (Fig. 2 ▸). That is, experimental data from unoriented H_II_ phases show no form factor minimum in this *q* range. The additional scattering may also explain the failure of the model-free analysis approach discussed above and possibly arises from packing defects between hexagonal bundles, for example at grain boundaries. However, surface-aligned H_II_ phases do not exhibit such scattering contributions (Ding *et al.*, 2004 ▸), disfavoring such a scenario. Hence, this appears to be only a property of unoriented H_II_ phases, fully immersed in aqueous solution. We speculate that the outermost boundary of H_II_ structures may try to avoid contact of the hydrocarbon with water by forming a lamellar layer, *i.e.* in some ways similar to hexosomes (Yaghmur & Glatter, 2009 ▸). Indeed, we were able to account for the additional diffuse scattering by adding a form factor of a laterally uniform, infinitely extended, planar bilayer
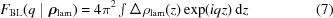
to the total scattered intensity, where *z* is the coordinate normal to the lamellar phase and 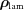 are the parameters describing the SLD of the lamellar phase. We cannot exclude that the additional diffuse scattering originates from unilamellar vesicles or other kinetically trapped aggregates formed during sample preparation.

Considering orientational averaging, we finally obtained for the total scattered intensity
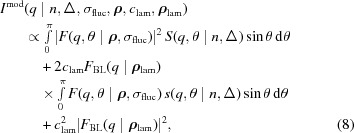
where 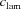 denotes the fraction of the lamellar phase. The structure factor 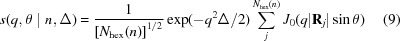
was derived analogously to the H_II_ structure factor [equation (2)] and the form factor is 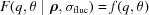
 × 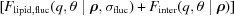 [see equation (3)].

### 3.2. Composition-specific modeling   

**Figure 3:**
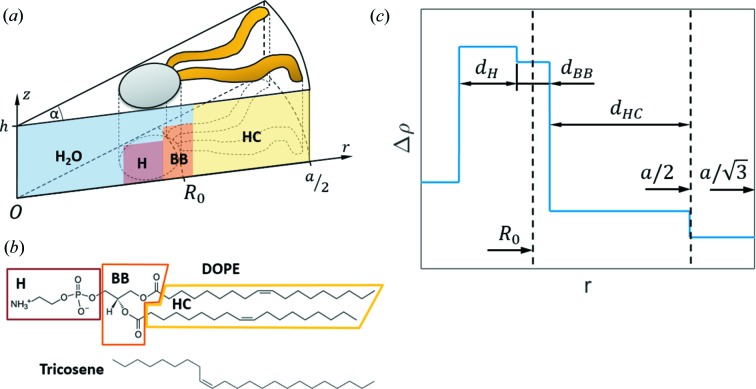
Composition-specific SLD modeling of phosphatidylethanolamines. (*a*) The unit cell of a single lipid has the shape of a cylinder sector of radius *a*/2. (*b*) Parsing of DOPE into head (H), backbone (BB) and hydrocarbon chain (HC) and chemical structure of tricosene. (*c*) Scheme of a corresponding electron density profile (see also Fig. 1).

In this section we develop a model for the SLDs described by the parameters 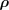 and 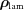. For increased structural fidelity we considered the minimum number of parameters. We also constrained the SLDs by the specific molecular compositions. Assuming that tricosene partitions exclusively into the interstitial space, the PE structure was parsed into three cylindrical shells of a wedge-shaped lipid unit cell of opening angle α and height *h* (Fig. 3 ▸): (i) the headgroup (H), consisting of phosphate and ethanolamine groups, (ii) the glycerol backbone (BB), given by the carbonyl and glycerol groups, and (iii) the tails (HC) consisting of all methyl, methine and methylene groups. The outer radius of the wedge *a*/2 is evaluated in advance from the Bragg peak positions 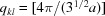
 × 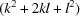, where *k* and *l* are the Miller indices. The position of the neutral plane *R*_0_ was assumed to be in the center of the BB shell. This was motivated by bending/compression experiments, which obtained estimates for the location of the neutral plane within the lipid backbone regime (Kozlov & Winterhalter, 1991 ▸; Leikin *et al.*, 1996 ▸). In our model, the entire PE structure is described by the intrinsic curvature *C*_0_ = −1/*R*_0_, the width of the headgroup 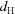 and the width of the backbone 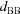. Further structural parameters of interest, such as the width of the hydrocarbon chain 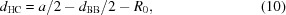
the lipid head-to-headgroup length 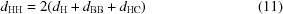
and the radius of the water core 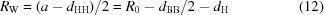
follow from these three parameters.

In the case of X-ray scattering the SLDs (electron densities) of each shell are given by ρ_*k*_ = *n* ^e^_*k*_/*V*_*k*_ with *k* ∈ {H, BB, HC}, where *n* ^e^_*k*_ is the number of electrons of a given quasi-molecular lipid fragment, *V*_H_ = 110 Å^3^, *V*_BB_ = 135 Å^3^ (Kučerka *et al.*, 2015 ▸) and *V*_HC_ = *V*_lipid_ − *V*_BB_ − *V*_H_. Furthermore, we estimated 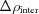 [equation (6)] of tricosene by molecular averaging over the fractional volumes of 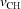, 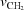 and 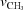 (Kučerka *et al.*, 2015 ▸) (see supporting Table S1). In our model the electron density is sufficiently described by the parameters *C*_0_, *d*_H_, *d*_BB_ and *V*_lipid_, and hence 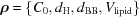. All other parameters can be deduced from these by using the lipid contribution to the volume of the *k*th shell: 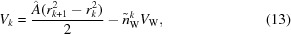
where 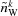 is the number of water molecules within each shell and *V*_W_ = 30 Å^3^ is the molecular volume of water. 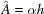 is the mantle area of a sector of unitary radius and can be obtained using equation (13) with *k* = HC and 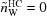: 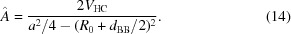
Hence, equation (13) also defines 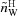 and 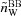.

Using our parametrization, it is straightforward to derive the area per lipid at any position within the molecule. For example, the area per lipid in the neutral plane is calculated as 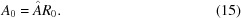
Furthermore, following Israelachvili (2011 ▸), the molecular shape parameter is given by 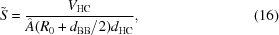
where 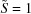 represents cylindrical (lamellar-phase-forming) molecules, and 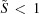 or 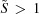 typify molecules inducing negative or positive monolayer curvatures, respectively. In particular, 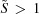 for amphiphiles forming aggregates with negative curvature, like the H_II_ structure.

The form factor of the additional lamellar phase was calculated by integrating equation (7), using a simple SLD model consisting of head and tail slabs (Székely *et al.*, 2010 ▸): 
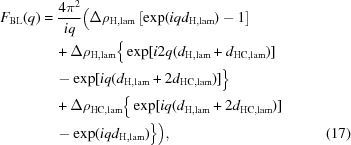
where 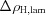 and 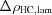 are the headgroup and hydrocarbon SLDs relative to water, respectively. These were derived as detailed above by counting the number of electrons in each slab and dividing by the corresponding volumes 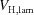 or 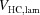. Assuming that 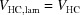 and 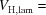 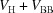, the hydrocarbon slab thickness results from 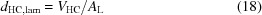
and the headgroup thickness from 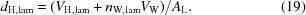
Hence, the area per lipid 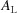 and the number of headgroup-associated water molecules 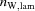 are the only parameters for the lamellar phase, 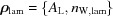.

## 4. Parameter estimation using Bayesian probability theory   

The final model for scattered intensities of unoriented fully hydrated H_II_ is given by
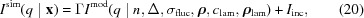
where *I* ^mod^ is given by equation (8), Γ is an instrumental scaling constant and *I*_inc_ accounts for incoherent scattering. In total, we have 12 model parameters, denoted by **x**, which are listed in Table 1 ▸.

There are various ways of estimating the parameters **x**. For a given data set **I** with the standard deviations 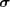 in the presence of a well defined global minimum the method of least squares yields fitting parameters by minimizing a cost function 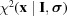. However, such an approach led for our present data to a significant variation of results between consecutive optimization runs, indicating a cost function landscape with a weakly defined global minimum. Thus, besides unreliable **x** values, also error estimates and potential correlations between the parameters remained undetermined.

To achieve higher confidence in our results we decided to use Bayesian probability theory; for a detailed introduction, see Jaynes & Bretthorst (2003 ▸), Gregory (2005 ▸), Sivia & Skillings (2012 ▸) and von der Linden *et al.* (2014 ▸). In brief, we were interested in deriving the probability 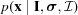, meaning the probability of the parameters **x** given the set of experimental data **I** with standard deviations 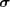 and additional information 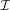 which might be present, such as the finite width of a lipid molecule. In the framework of Bayesian probability theory, Bayes’ theorem shows how to calculate this quantity, also called the posterior:
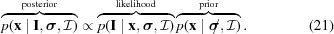
Bayes’ theorem constitutes the rule for learning from experimental data. The prior probability 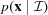 represents the prior knowledge about the unknown quantities **x**. We crossed out 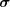 in the prior, since the prior does not depend on the standard deviations of the data. The likelihood 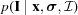, representing the probability for the data **I** given **x** and 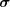, includes all information about the measurement itself. The prior probabilities 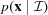 were assumed to be uniformly distributed between lower 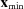 and upper 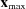 constraints for all parameters. Therefore, 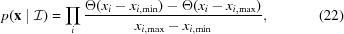
where Θ(*x*) is the Heaviside step function. For each parameter, *x*_*i*_, *x*_*i*,min_ and *x*_*i*,max_ denote physically meaningful boundaries. In particular, we constrained the parameters 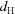 and 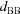 by the conditions 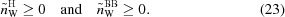
This means that the volumes of the head and backbone shell [equation (13)] have to be large enough to accommodate the respective molecular group.

We consider the likelihood 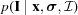. Since we did not trust the experimentally derived error estimates 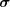 for the scattered intensities, we assumed that their real values 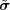 are connected to 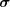 by a scaling factor η. Using the marginalization rule of Bayesian probability theory we obtain
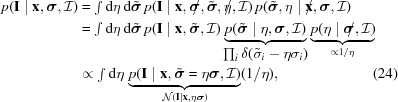
where 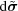 is shorthand for 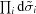. Here, we have specifically made use of the Jeffreys prior *p*(η) ∝ 1/η (Jeffreys, 1946 ▸), where η is a scaling invariant, meaning that we have *a priori* no idea about the order of magnitude of η. This scaling connects the likelihood [equation (24)] to the multivariate Gaussian:
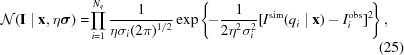
where η has to be integrated out, respecting the Jeffreys prior, and *I*_*i*_^obs^ denotes the observed intensity at *q*_*i*_. Here, *N*_*q*_ is the number of data points for a given scattering pattern.

For illustration, consider an arbitrary function 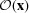 with the parameters **x**. The expectation value of 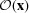 is then calculated by evaluating the integral
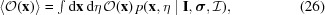
where
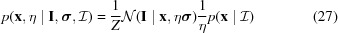
with the normalization constant *Z*. For example, using 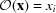 produces the expectation value 〈*x*_*i*_〉 for parameter *x*_*i*_.

A suitable technique for performing these integrals and sampling from the probability distribution 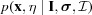 is Markov chain Monte Carlo (MCMC), which is based on constructing a Markov chain with the desired distribution of **x** in equilibrium. We used the Metropolis–Hastings algorithm for generating the Markov chain {**x** ^*k*^, η^*k*^}. Starting with a parameter set **x** ^*k*=1^ and η^*k*=1^, every new parameter set *k* + 1 can be proposed by varying parameters in the old parameter set *k*. The new parameter set *k* + 1 is accepted with the probability
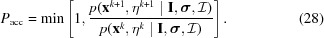
The first 10−20% of a Markov chain has to be discarded to ensure that the rest of the Markov chain is independent of the initial state **x** ^*k*=1^ and η^*k*=1^, *i.e.* the Markov chain is equilibrated to the desired distribution.

In addition, the states in the Markov chain have to be uncorrelated, which can be ensured by taking only every *N*_run_th state of the Markov chain. *N*_run_ can be controlled by evaluating the autocorrelation function or using techniques like binning and jackknifing. Finally, the observable can be estimated by
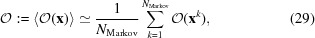

*i.e.* taking the mean value of *N*_Markov_ uncorrelated Markov chain elements. The confidence intervals can be estimated from 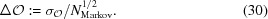
The variance
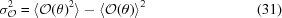
can in turn be estimated from the Markov chain. Alternatively, the uncertainty can be determined from independent MCMC runs.

Since the Markov chain {**x** ^*k*^, η^*k*^} is a representative sample drawn from 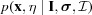 it can be used to plot the probability distribution, for example the marginal probability distribution 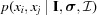 for the parameters *i* and *j* by plotting the two-dimensional histogram of the samples {*x* ^*k*^_*i*_} and {*x* ^*k*^_*j*_}. This allows us to unravel correlations between the parameters *i* and *j*, *i.e.* it allows the analysis of mutual parameter dependencies that could lead to ambiguous results using the least-squares method. Additionally, the cost function 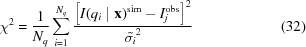
is saved for every run.

## 5. Results and discussion   

### 5.1. Tests of the analysis   

We first explored our model and the Bayesian analysis on the well studied H_II_ structure of DOPE (Turner & Gruner, 1992 ▸, 1989 ▸; Harper *et al.*, 2001 ▸; Kollmitzer *et al.*, 2013 ▸). We emphasize that the choice of our model restricts the algorithm to a certain functional space for describing the scattering pattern. This constraint can lead to some systematic deviations from the experimental data, and Bayesian model comparison can be used for choosing the appropriate model.

**Figure 4:**
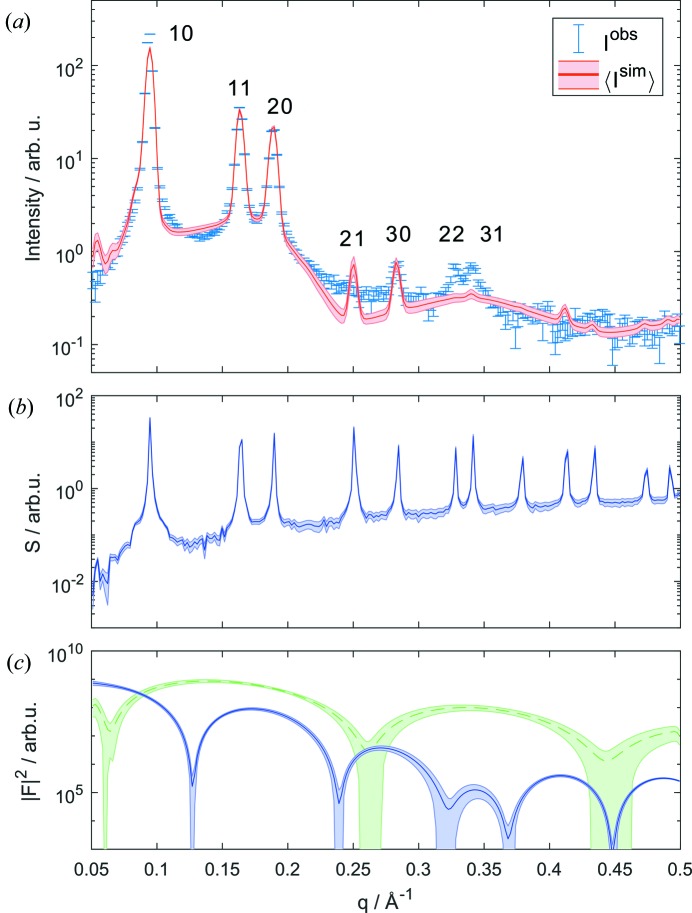
Expectation value and error bands of the intensity of fully hydrated DOPE at 308 K (*a*), and the involved structure (*b*) and form factors (*c*) (blue: hexagonal form factor; green: lamellar form factor).

Clearly, our model is able to account well for most features of the scattering pattern up to *q* ≃ 0.5 Å^−1^ (Fig. 4 ▸). In particular, the bilayer form factor compensates well for the form factor minimum between the 10 and 11 peaks, but adds also some diffuse scattering at higher *q* values. The small peak observed in the calculated intensity at very low *q* is an artifact resulting from the structure factor. This could be removed by averaging over a distribution of domains (Freiberger & Glatter, 2006 ▸), but does not affect the overall structural results and has therefore been omitted to reduce computational cost (see also above). Furthermore, the proximity of a form factor minimum to the 21 peak of the H_II_ phase nearly causes an extinction in the scattering data. Note that tricosene-free DOPE samples exhibit a clear 21 reflection [Fig. S4(*c*)]. However, because of strain-induced distortions of the hexagonal prisms such samples cannot be analyzed with the present model. The maximum aposterior (MAP) solution still shows a slightly more pronounced 21 peak, since a perfect fit in this *q* range would lead to significant deviations between the model and experimental data close to the 20 peak, which due to its smaller errors has a higher significance in contributing to the overall goodness of the MAP solution. Additionally, our MAP solution underestimates the contributions of the 22 and 31 peaks owing to the proximity of the cylinder form factor to two minima. To account for this we tested SLD models of greater complexity, by considering either a separate slab for the methyl terminus of the hydrocarbon chain or a linear decrease of the electron density in the hydrocarbon regime. However, this did not lead to a significant improvement of the agreement between the model and the experimental data in this *q* range. In order to avoid overfitting, we therefore remained with the SLD model as described in Section 3. Table S2 lists the corresponding expectation values 〈**x**〉 and variances σ_**x**_. In order to check for reproducibilty, we prepared a fresh DOPE sample. The results listed in Table S2 show that all structural lipid parameters are identical within experimental uncertainty [see also Fig. S4(*b*)]. Differences in lattice parameters, such as *a* and Δ, relate to slight variations of tricosene content.

**Figure 5:**
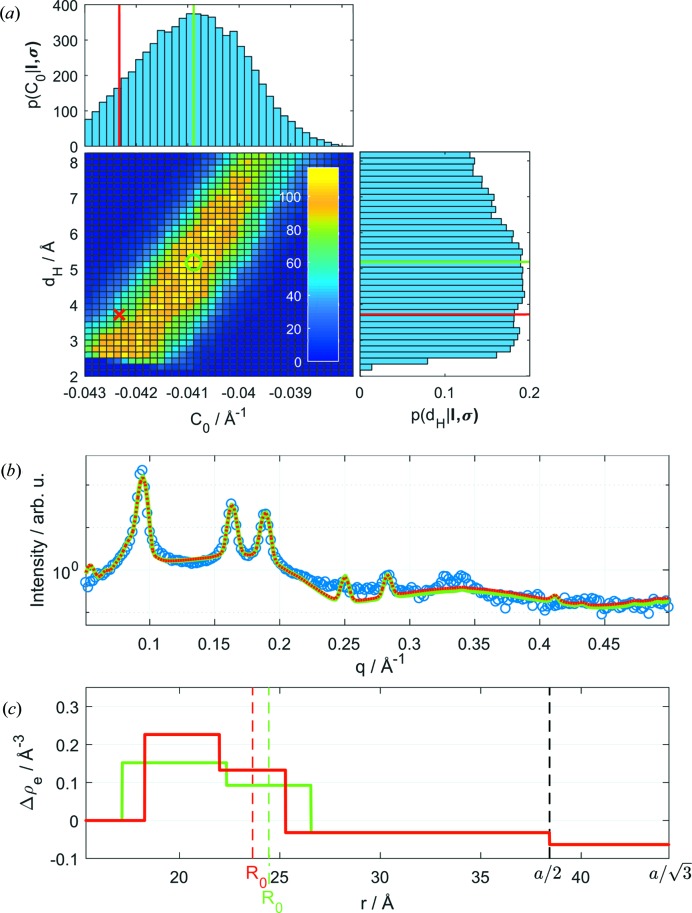
Marginal posterior distributions 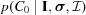 and 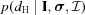 of intrinsic curvature *C*_0_ and headgroup width *d*_H_ and 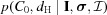 (*a*). The red cross and corresponding lines mark the sample with the lowest χ^2^ (MAP solution), and the green circle shows the mean value of the distribution. Panels (*b*) and (*c*) show the corresponding fits and SLD profiles.

One of the benefits of the Bayesian analysis compared with the least-squares method is the possibility to reveal correlations between adjustable parameters, just by looking at the 2D marginal probability density distributions [see *e.g.* Fig. 5 ▸(*a*)]. Marginal distributions of all other parameters are shown in the supplementary Figs. S1–S3. Most parameter pairs show no correlations and exhibit probability distributions with Gaussian-like behavior, including σ_fluc_, *V*_lipid_ and Γ. Significant correlations can be seen for the parameters *d*_H_ and *d*_BB_ with *C*_0_, as well as between *n*_W,lam_ and *A*_L_. A strong correlation between two parameters suggests the possibility to simplify the model. However, this would be highly specific for a given amphiphile and was consequently not considered. The parameters *d*_H_ and *d*_BB_ exhibit broad probability distributions with no well defined maximum. In turn *C*_0_ has a peaked probability distribution yielding a well defined estimate value and uncertainty.

Here, we discuss for illustration purposes the correlation between *C*_0_ and *d*_H_ [Fig. 5 ▸(*a*)]. Solutions along the diagonal line give similar scattering intensities, but lead to significantly different electron density profiles [see Figs. 5 ▸(*b*) and 5 ▸(*c*)].

The correlation between *d*_H_ and *C*_0_ may appear counterintuitive. From geometric/physical arguments it follows that small *d*_H_ values represent a bending of the phosphate–ethanolamine director of the lipid headgroup toward the polar/apolar interface, which leads to a shift of *C*_0_ toward more positive values. However, this would lead to significantly different scattered intensities and hence to non-optimal solutions. The mathematical algorithm therefore aims to com­pen­sate for this by decreasing *C*_0_ for small *d*_H_. The abrupt drop of the headgroup thickness probability distribution at small *d*_H_ is due to the termination criterion [equation (23)].

In the following, we discuss some expectation values 〈**x**〉 and the errors σ_**x**_ obtained by applying equations (29) and (31). Table 2 ▸ compares the obtained structural parameters of DOPE with existing literature values. Our results are in good agreement with previous reports, given the different additives (alkanes or alkenes, some did not use any filler molecule) and slight variations in temperatures. Note that in some cases *A*_0_ and *C*_0_ have been reported for the pivotal plane. The pivotal plane marks the position within the lipids where the molecular area does not change upon deformation and is usually slightly closer to the hydrocarbon tail than the neutral plane (Leikin *et al.*, 1996 ▸; Kollmitzer *et al.*, 2013 ▸). This leads to a slight shift of *C*_0_ toward positive values.

The most direct comparison of *C*_0_ can be made with our previous work (Kollmitzer *et al.*, 2013 ▸), which was performed at the same temperature and tricosene content. Here, we find that the global model combined with Bayesian analysis yields an intrinsic curvature, which agrees within experimental uncertainty with our previous result.

### 5.2. Effect of temperature   

**Figure 6:**
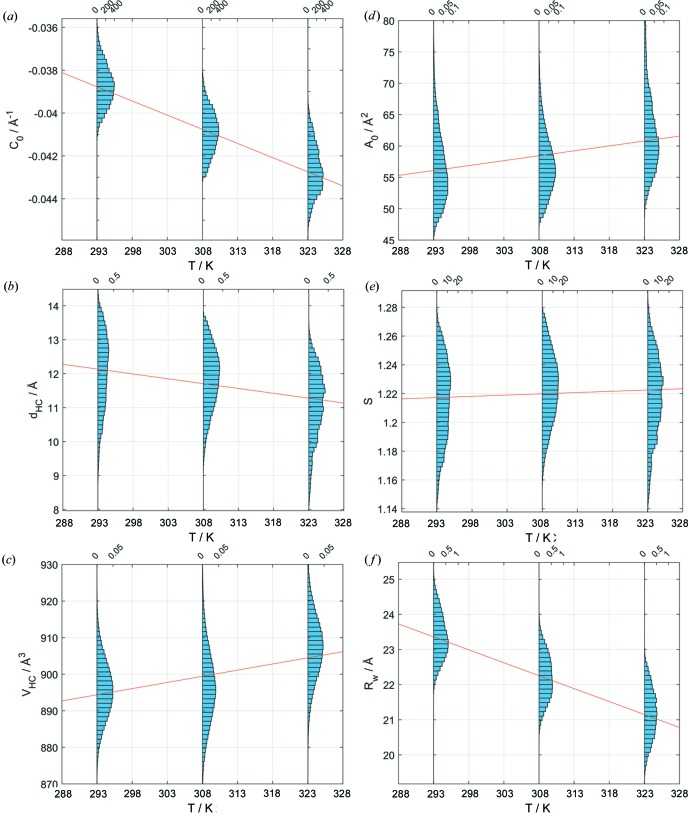
Structural parameters of DOPE H_II_ as a function of temperature resulting from the Bayesian analysis. Probability densities (*a*) of the intrinsic curvatures, (*b*) of the hydrocarbon chain length, (*c*) of the hydrocarbon chain volume, (*d*) of the area per lipid in the neutral plane, (*e*) of the shape parameter and (*f*) of the radius of the water cylinder. Red lines indicate linear regressions of the probability density distributions.

Increasing the temperature for DOPE should yield a decrease of lipid chain length and a concomitant significant increase of the area per lipid at the methyl terminus leading to more negative intrinsic curvatures as reported previously (Turner & Gruner, 1992 ▸, 1989 ▸; Harper *et al.*, 2001 ▸; Kollmitzer *et al.*, 2013 ▸). Indeed, our analysis yielded a linear decrease of *C*_0_ and 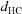 (Fig. 6 ▸). The slope Δ*C*_0_/Δ*T* = (−1.323 ± 0.001) × 10^−4^ (Å K)^−1^ is identical to our previously reported value (Kollmitzer *et al.*, 2013 ▸). The relative change of the chain length is in turn 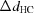/Δ*T* = (−0.0188 ± 0.0001) Å K^−1^. Interestingly, the shape parameter 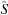 shows only a modest increase of 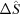/Δ*T* = (1.75 ± 0.03) × 10^−4^ K^−1^, despite the more negative *C*_0_ values at higher temperatures and despite the decrease of 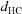 and the increase of 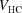 [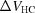/Δ*T* = (0.3364 ± 0.0010) Å^3^ K^−1^]. This results from a concomitant increase of headgroup area [Δ*A*_0_/Δ*T* = (0.1567 ± 0.0006) Å^2^ K^−1^] in the neutral plane – and analogously also at the position of the polar/apolar interface 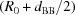 – which compensates for the changes of 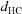 and 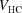. The radius of the water core decreases [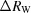/Δ*T* = (−7.364 ± 0.007) × 10^−2^ Å K^−1^].

## 6. Effect of hydrocarbon chain composition   

Finally, we tested the applicability of the analysis technique to PEs with differing hydrocarbon chain composition. In particular, we studied the H_II_ phases of POPE, which has a palmitoyl and an oleoyl chain, DMPE, which has two myristoyl chains, and diC16:1PE, with two palmitoleoyl hydrocarbons. Note that pure POPE forms an H_II_ phase only above 344 K, while the lamellar-to-H_II_ phase-transition temperature *T*_H_ for pure di16:1PE was reported to be 316.6 K and *T*_H_ > 373 K for DMPE (Koynova & Caffrey, 1994 ▸). The addition of alkanes or alkenes to inverted hexagonal phases is known to reduce stress resulting from interstitial space between the individual rods (Vacklin *et al.*, 2000 ▸; Chen & Rand, 1998 ▸; Kirk & Gruner, 1985 ▸). We previously demonstrated that tricosene sufficiently lowers *T*_H_ for POPE to perform an H_II_ phase analysis at physiological temperature (Kollmitzer *et al.*, 2013 ▸). Similarly, di16:1PE formed a neat H_II_ phase at 308 K upon addition of 12 wt% tricosene (see below). In the case of DMPE we found a pure H_II_ scattering pattern only for *T* ≥ 353 K, indicating a significantly less negative *C*_0_. For this reason we performed the global analysis at 308 K for POPE and di16:1PE and at 353 K for DMPE.

**Figure 7:**
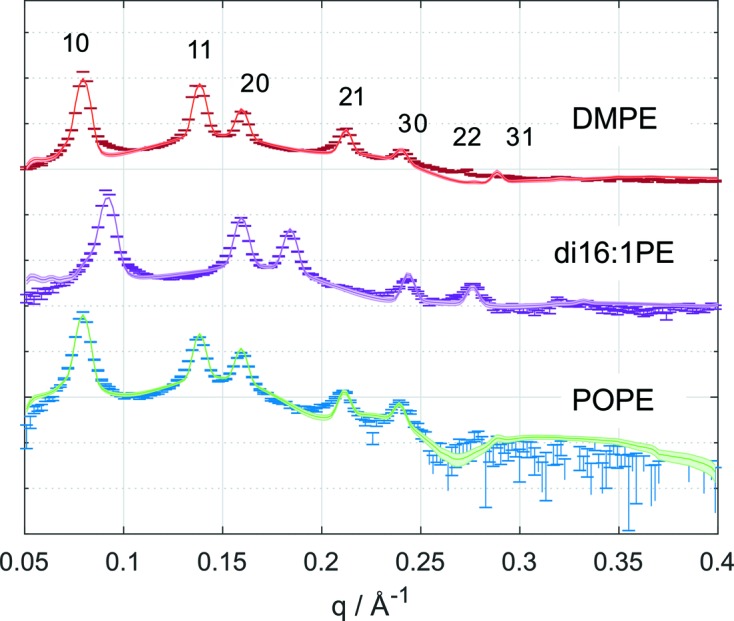
Data points (including error bars) and expectation values of the intensity (with error bands) of SAXS patterns of POPE (308 K), di16:1PE (308 K) and DMPE (353 K).

**Figure 8:**
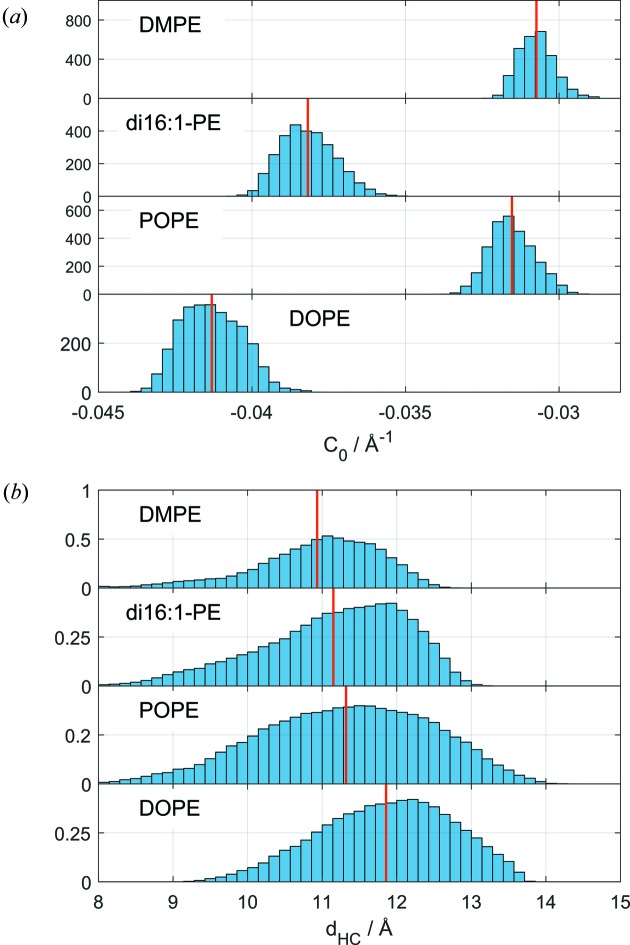
Intrinsic curvature (*a*) and hydrocarbon chain length (*b*) probability densities and mean values (red lines) for various lipids at 308 K (except DMPE: 353 K).

In contrast to the results for DOPE, the 21 peak was clearly present in the scattering data of all three lipids, a feature which helped us to obtain a better agreement of the model with experimental data (Fig. 7 ▸). The corresponding probability density distributions for *C*_0_ clearly show that monounsaturated hydrocarbons induce significantly more negative intrinsic curvature than saturated hydrocarbons, which is due to the kink induced by the *cis* double bond. The proximity of values for POPE and DMPE is attributed to the temperature difference and the associated decrease of *C*_0_ (Fig. 8 ▸). Assuming a similar temperature dependence to that observed for DOPE yields *C*_0_ close to zero for DMPE at 308 K, which agrees with the well established observation that DMPE prefers to form bilayers at ambient temperatures. Besides the difference between saturated and unsaturated hydrocarbons, our analysis also clearly shows that 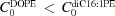. That is, increasing the chain length of monounsaturated acyl chains also leads to a more negative *C*_0_ value. This signifies that the kink induced by the *cis* double bond leads to a progressive increase of hydrocarbon splay upon acyl chain extension.

The mean values of hydrocarbon chain length show a trend in the expected direction, *i.e.* they increase with the number of hydrocarbons (Fig. 8 ▸), but all cases exhibit a broad distribution as a result of the not well defined backbone width 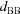.

The expectation values for *C*_0_ and 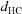 for the different lipids are listed in Table 3 ▸, including resulting structural parameters for 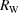, *A*_0_, 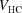 and 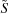. Previously, we reported *C*_0_ = −0.0316 Å^−1^ for POPE at 310 K (Kollmitzer *et al.*, 2013 ▸), which is in excellent agreement with our present value. Regarding other structural parameters, in particular we found that 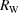 and *A*_0_ decrease as *C*_0_ becomes more negative, which is mainly attributed to the geometry of the H_II_ phase. The hydrocarbon chain volumes are in agreement with the chemical compositions. That is, DMPE with two C14:0 chains has the smallest and DOPE with two C18:1 chains has the largest 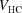 value, and the volumes of POPE and diC16:1PE take intermediate values. Our hydrocarbon volume of POPE is about 4% lower than the value reported for POPE in the absence of tricosene at the same temperature, where it forms a fluid lamellar phase (Kučerka *et al.*, 2015 ▸). This indicates a slightly tighter hydrocarbon chain packing in fully relaxed monolayers. The shape parameter (DMPE ≃ POPE < diC16:1PE < DOPE) clearly shows that among all lipids presently studied DOPE has the highest propensity to form an H_II_ phase, which is consistent with its low 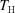 (Koynova & Caffrey, 1994 ▸).

## 7. Conclusions   

We have introduced a global scattering model for fully hydrated, unoriented H_II_ phases. Compared with previous models for H_I_ phases (Freiberger & Glatter, 2006 ▸; Sundblom *et al.*, 2009 ▸), the H_II_ phase analysis required the addition of diffuse scattering not originating from hexagonal structures. While the exact origin of this additional contribution remains unclear, we successfully modeled the measured SAXS pattern upon including a lamellar form factor. The SLD of the lipid unit cell was constrained by compositional modeling using complementary information on lipid volume and structure. This description is generic and entails the analysis of SAXS and small-angle neutron scattering (SANS) data. In particular a joint analysis of SAXS and differently contrasted SANS data [see *e.g.* Pabst *et al.* (2010 ▸) and Heberle & Pabst (2017 ▸)] might be beneficial for increased structural resolution regarding the lipid head and backbone groups.

Here, we analyzed SAXS data using Bayesian probability theory combined with MCMC simulations. This was necessary because of the weakly defined global minimum of the optimization cost function. The full probabilistic approach provides the probability density distributions of the involved parameters, leading to reliable parameter estimates including errors.

The obtained estimates are in good agreement with previously reported structural data of DOPE and POPE. We further provided details for lipid structures of DMPE and di16:1PE in the H_II_ phase, clearly demonstrating that out of all of the presently studied lipids DMPE is the least prone to form an H_II_ phase. The developed technique will be easily transferred to other H_II_ phase amphiphiles using appropriate compositional modeling. In particular, we envisage a high potential for applications in drug-delivery formulations involving H_II_ structures that exhibit only weak Bragg peaks but significant contributions from diffuse scattering, such as hexosomes [see *e.g.* Yaghmur & Glatter (2009 ▸)]. Another potential application is the determination of intrinsic lipid curvatures of lamellar-phase-forming lipids using mixtures with DOPE (Kollmitzer *et al.*, 2013 ▸), which is particularly encouraged by the high robustness of the retrieved *C*_0_ estimates. Such approaches are currently being explored in our laboratory.

## Supplementary Material

Supporting information (4 figures, 3 tables). DOI: 10.1107/S1600576719002760/vg5104sup1.pdf

## Figures and Tables

**Table 1:** Overview of the model parameters for fully hydrated, unoriented H_II_ phases

Occurrence	*x*	Meaning
Structure factor	Δ	Mean-square displacement of the lattice points
	*n*	Number of hexagonal shells (domain size)

H_II_ form factor	σ_fluc_	Fluctuation constant of the lipid unit cell
	*C*_0_	Intrinsic curvature
	*d*_H_	Width of the lipid headgroup
	*d*_BB_	Width of the lipid backbone
	*V*_lipid_	Lipid volume

Lamellar form factor	*c*_lam_	Lamellar form factor scaling constant
	*A*_L_	Area per lipid of the lamellar phase
	*n*_W,lam_	Number of water molecules in the headgroup slab of the lamellar phase

Signal scaling	Γ	Instrumental scaling constant
	*I*_inc_	Incoherent background

**Table 2:** Comparison of structural parameters of DOPE with literature values

*a* (Å)	*V*_L_ (Å^3^)	*d*_HH_ (Å)	*R*_W_ (Å)	*A*_0_ (Å^2^)	*C*_0_ (Å^−1^)	Reference
76.9 ± 0.2	1142 ± 10	32.4 ± 1.3	22.2 ± 0.7	62.2 ± 6.0	−0.0409 ± 0.0010	This work
71.9	1224	31.8	20.0	–	–	Tate & Gruner (1989 ▸)^*a*^
74.9	–	–	22	–	−0.033^*i*^	Rand *et al.* (1990 ▸)^*b*^
72.75	–	–	19.1	–	–	Turner & Gruner (1992 ▸)^*c*^
–	–	–	–	–	−0.0367 ± 0.0005	Leikin *et al.* (1996 ▸)^*d*^
72.9	1220	36.0	20.4	47.4^*i*^	–	Harper *et al.* (2001 ▸)^*e*^
76	–	–	–	51.5^*i*^	−0.031^*i*^	Di Gregorio & Mariani (2005 ▸)^*f*^
–	–	–	–	–	−0.0399 ± 0.0005	Kollmitzer *et al.* (2013 ▸)^*g*^
–	–	–	–	–	−0.0365 ± 0.0012	Chen *et al.* (2015 ▸)^*h*^

(*a*) *T* = 308 K, dodecane. (*b*) *T* = 295 K, tetradecane. (*c*) *T* = 303 K, dodecane. (*d*) *T* = 298 K. (*e*) *T* = 303 K. (*f*) *T* = 298 K. (*g*) *T* = 308 K, tricosene. (*h*) *T* = 303 K, tetradecane. (*i*) Determined in the pivotal plane.

**Table 3:** Comparison of structural parameters of different phosphatidylethanolamines

	*C*_0_ (Å^−1^)	*d*_HC_ (Å)	*R*_W_ (Å)	*A*_0_ (Å^2^)	*V*_HC_ (Å^3^)	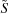
DMPE^*a*^	−0.0314 ± 0.0006	10.9 ± 0.9	30.6 ± 0.7	59 ± 6	737 ± 7	1.16 ± 0.02
di16:1PE^*b*^	−0.0382 ± 0.0009	11.1 ± 1.0	24.0 ± 0.7	60 ± 7	797 ± 8	1.20 ± 0.03
POPE^*b*^	−0.0317 ± 0.0007	11.3 ± 1.2	29.2 ± 0.9	68 ± 9	884 ± 9	1.17 ± 0.03
DOPE*^b^*	−0.0409 ± 0.0010	11.9 ± 0.9	22.2 ± 0.7	62 ± 6	897 ± 10	1.22 ± 0.03

(*a*) *T* = 353 K. (*b*) *T* = 308 K.
