# Supplementary material for: Global small-angle scattering data analysis of inverted hexagonal phases
Source: J Appl Crystallogr. 2019 Mar 28;52(Pt 2):403–14. doi: 10.1107/S1600576719002760 (PMC6448687; doi:10.1107/S1600576719002760)
Supplement: Supplementary file 1 [file j-52-00403-sup1.pdf]

Supplementary Material

Global Small-Angle Scattering Data Analysis of Inverted Hexagonal Phases

Moritz Frewein, Michael Rumetshofer, Georg Pabst

Content:

Fig. S1: Parameter Correlations 1
Fig. S2: Parameter Correlations 2
Fig. S3: Parameter Correlations 3
Fig. S4: Reproducibility Check; Sample without filler
Tab. S1: Electron density of tricosene
Tab. S2: Structural parameters of DOPE
Tab. S3: Structural parameters of di16:1PE, POPE, DMPE

1 Supplementary Figures

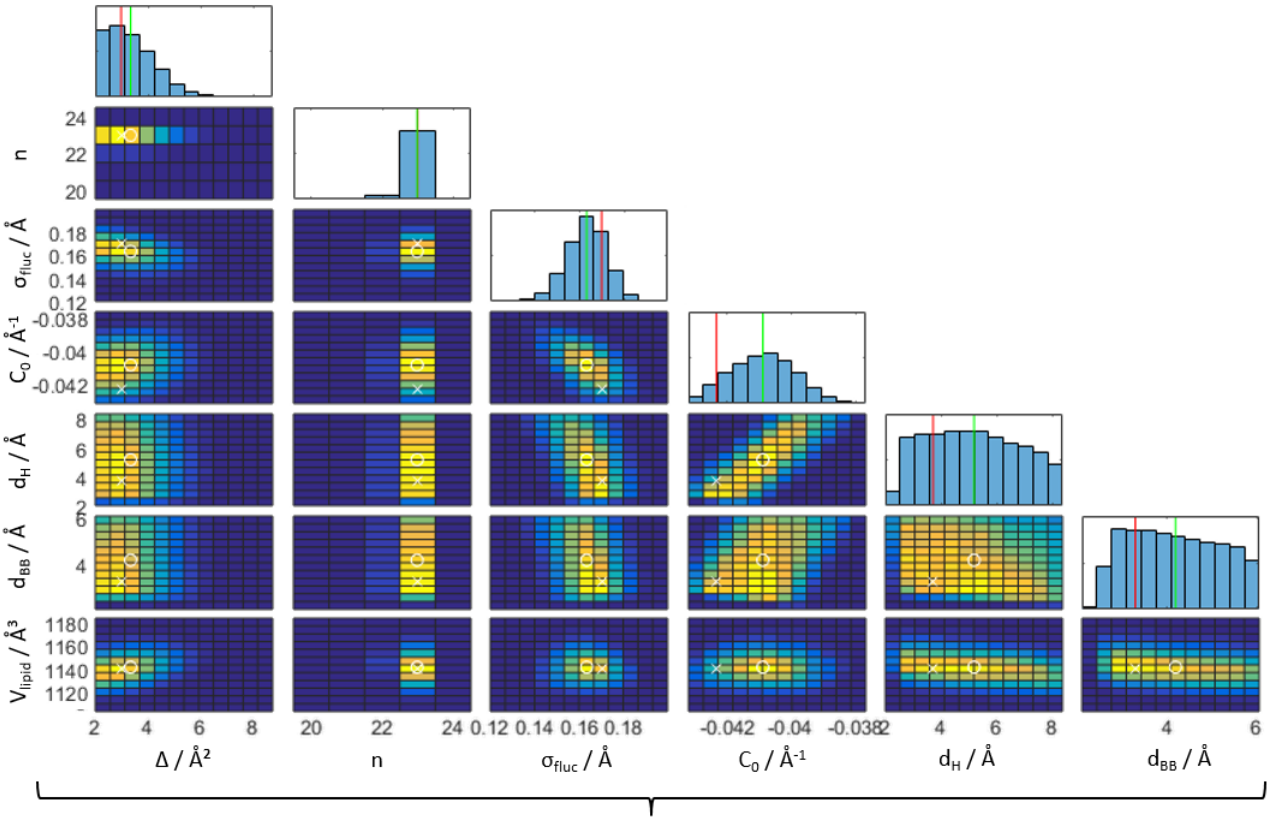

Fig. S2

Figure S1: Pairwise correlations between V_{lipid}, d_{BB}, d_{H}, C_0, σ_{fluc}, Δ, and n. The white crosses mark the samples with the lowest χ^2 (MAP solution), the white circles show the mean value of the distribution.

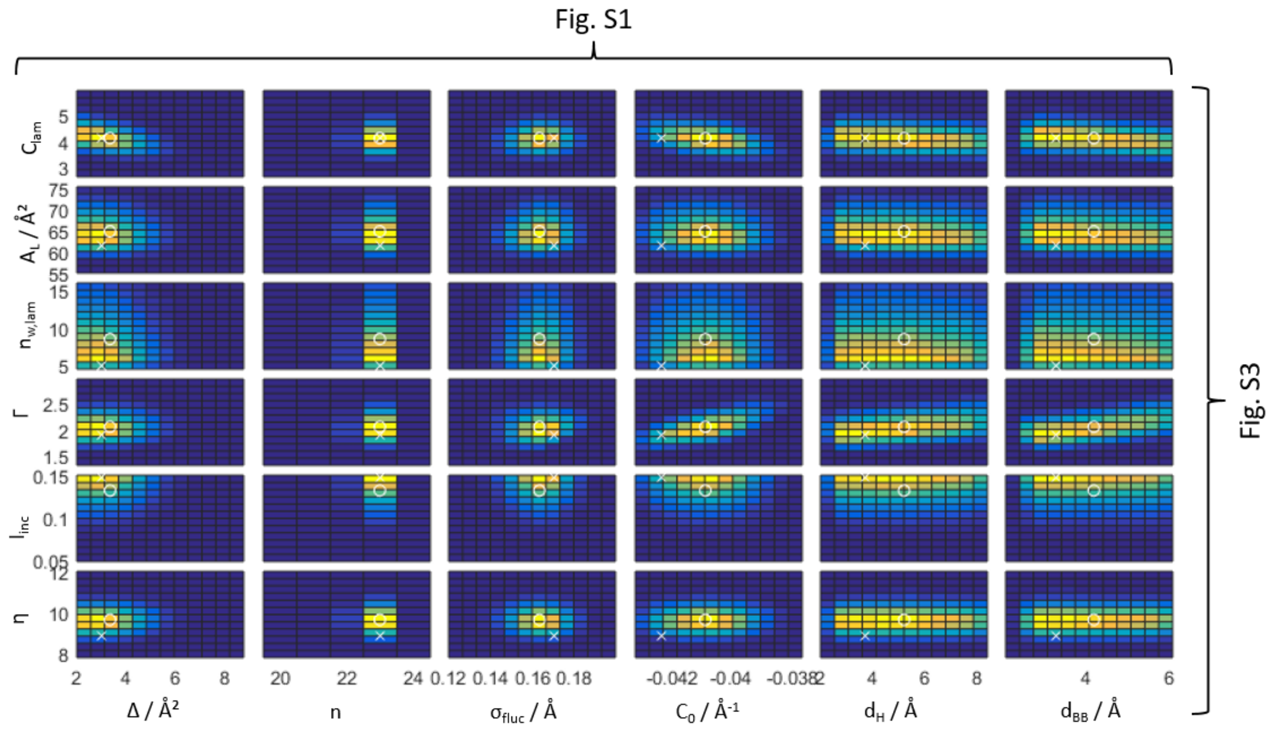

Figure S2: Pairwise correlations between η, $I_{\rm inc}$, Γ, $n_{\rm W,lam}$, $A_{\rm L}$, $c_{\rm lam}$, Δ, n, $\sigma_{\rm fluc}$, C_0, $d_{\rm H}$ and $d_{\rm BB}$. The white crosses mark the samples with the lowest χ^2 (MAP solution), the white circles show the mean value of the distribution.

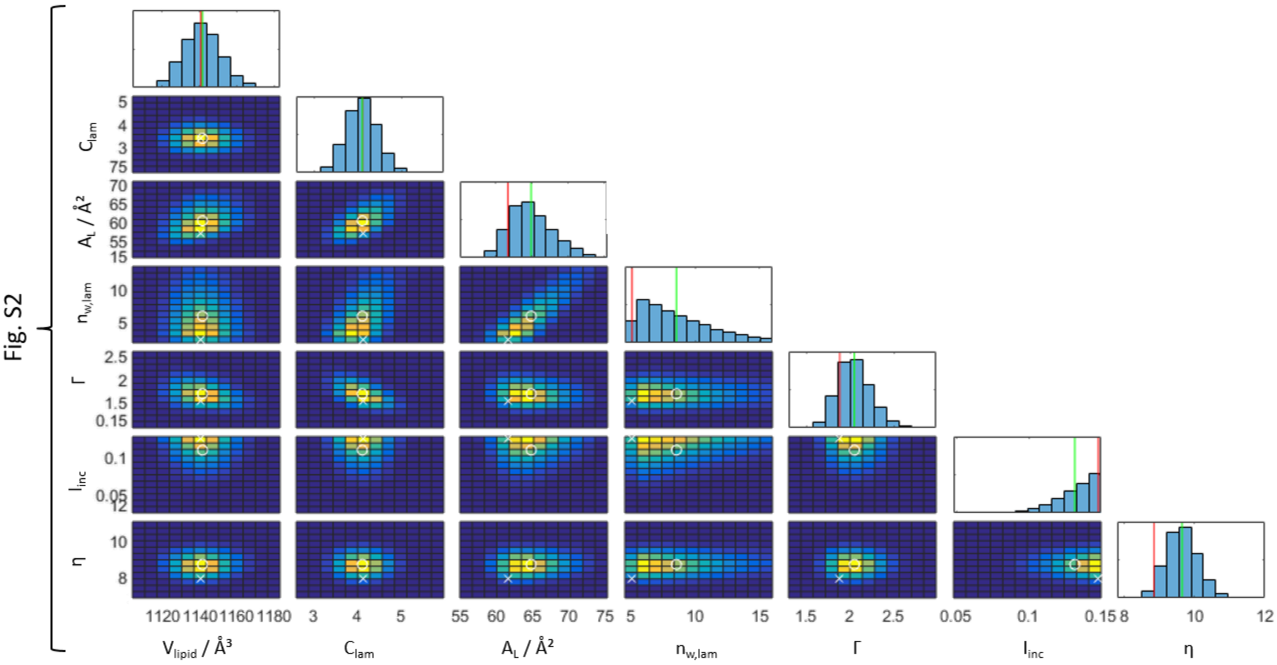

Figure S3: Pairwise correlations between η, I_{inc}, Γ, $n_{\text{W,lam}}$, A_{L}, c_{lam} and V_{lipid}. The white crosses mark the samples with the lowest χ^2 (MAP solution), the white circles show the mean value of the distribution.

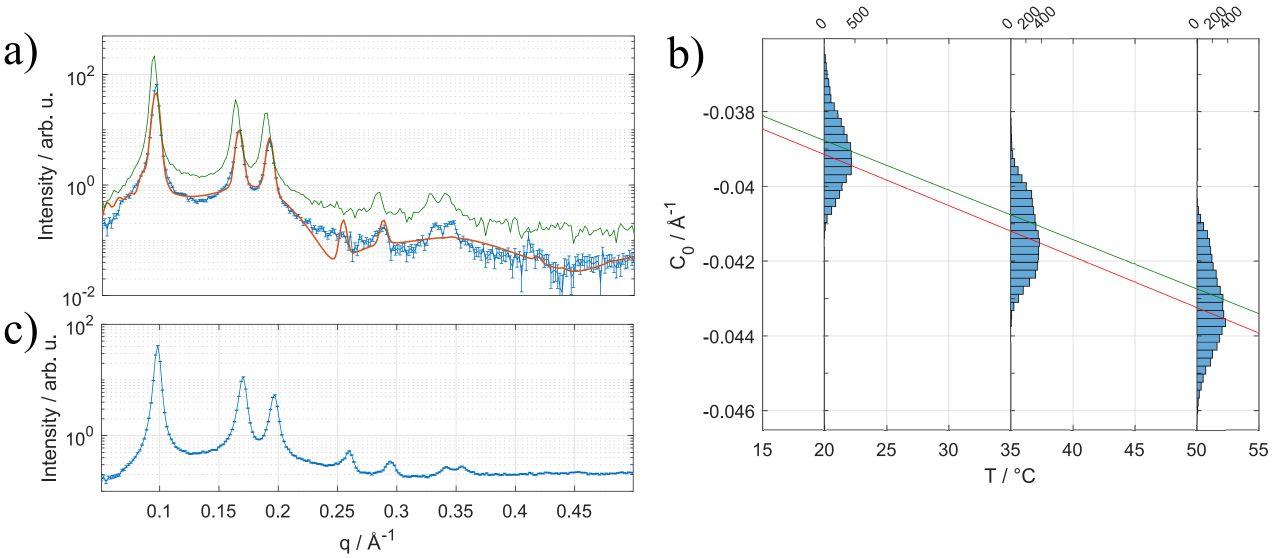

Figure S4: To check for reproducibility, we independently prepared a second sample of DOPE containing 12 wt.% tricosene. Panel a) shows the comparison of SAXS-data of the first (green) and the second (blue) DOPE sample and its MAP solution (orange), measured at 35°C. The marginal posterior distributions for C_0 of the second sample have a similar temperature dependence (red) as the first sample (green) (panel b). SAXS data in panel c) correspond to tricosene-free DOPE at 35 °C. In contrast to data presented in panel a) the (2,1) peak is clearly observed.

2 Supplementary Tables

Table S1: Electron density estimates of tricosene using fractional volumes reported in [1].

T / °C	ρ_{tric} / Å^{-3}
20	0.281
35	0.270
50	0.259
80	0.236

Table S2: $\langle \mathbf{x} \rangle$ and $\sigma_{\mathbf{x}}$ for DOPE from Bayesian analysis.

	$T = 20$°C	$T = 35$°C^a	$T = 50$°C
a / Å^b	80.4 ± 0.2	76.9 ± 0.2 (75.6 ± 0.2)	73.5 ± 0.2
Δ / Å^2	2.9 ± 1.1	3.3 ± 1.0 (6.6 ± 1.2)	6.7 ± 1.1
n	18 ± 1	23 ± 1 (20 ± 1)	29 ± 1
σ_{fluc} / Å	0.163 ± 0.009	0.163 ± 0.009 (0.166 ± 0.007)	0.176 ± 0.008
C_0 / Å^{-1}	-0.0387 ± 0.0010	-0.0409 ± 0.0010 (-0.0413 ± 0.0010)	-0.0426 ± 0.0011
d_{H} / Å	4.8 ± 1.7	5.2 ± 1.7 (4.5 ± 1.7)	5.1 ± 1.8
d_{BB} / Å	4.6 ± 1.3	4.2 ± 1.0 (4.1 ± 1.2)	4.2 ± 1.2
V_{lipid} / Å^3	1141 ± 8	1142 ± 10 (1156 ± 9)	1152 ± 9
c_{lam}	393 ± 33	411 ± 35 (413 ± 34)	387 ± 31
A_{L} / Å^2	61.1 ± 2.7	64.9 ± 3.1 (66.2 ± 3.2)	65.4 ± 2.7
$n_{\text{W,lam}}$	6.4 ± 2.6	8.5 ± 2.7 (8.5 ± 3.3)	6.2 ± 2.6
Γ	2.08 ± 0.22	2.05 ± 0.19 (0.75 ± 0.07)	2.21 ± 0.21
I_{inc}	0.133 ± 0.014	0.132 ± 0.015 (0.033 ± 0.009)	0.135 ± 0.012
η	8.18 ± 0.39	9.65 ± 0.46 (8.16 ± 0.39)	7.13 ± 0.34

^{a} values in brackets correspond to an independently prepared sample (Fig. S4)
^{b} determined from peak positions

Table S3: $\langle \mathbf{x} \rangle$ and $\sigma_{\mathbf{x}}$ for di16:1 PE, POPE and DMPE from Bayesian analysis

	di16:1 PE, 35 °C	POPE, 35 °C	DMPE, 80 °C
a / Å[a]	78.8 ± 0.2	90.8 ± 0.2	90.7 ± 0.2
Δ / Å^2	4.8 ± 1.7	17.3 ± 1.6	8.4 ± 1.5
n	18 ± 1	24 ± 1	29 ± 1
σ_{fluc} / Å	0.167 ± 0.010	0.141 ± 0.009	0.131 ± 0.011
C_0 / Å^{-1}	-0.0382 ± 0.0009	-0.0315 ± 0.0008	-0.0307 ± 0.0006
d_{H} / Å	4.5 ± 1.7	5.2 ± 2.1	5.1 ± 1.8
d_{BB} / Å	4.1 ± 1.2	4.7 ± 1.4	3.7 ± 1.0
V_{lipid} / Å^3	1042 ± 8	1129 ± 9	982 ± 7
c_{lam}	451 ± 43	517 ± 36	706 ± 46
A_{L} / Å^2	64.2 ± 2.2	63.0 ± 1.5	63.4 ± 1.5
$n_{\mathrm{W,lam}}$	13.0 ± 1.7	10.8 ± 1.1	11.2 ± 0.7
Γ	1.42 ± 0.16	0.72 ± 0.08	2.53 ± 0.27
I_{inc}	0.264 ± 0.023	0.010 ± 0.009	0.930 ± 0.042
η	7.72 ± 0.37	5.42 ± 0.31	12.50 ± 0.60

[a] determined from peak positions

References

[1] N. Kučerka, B. van Oosten, J. Pan, F.A. Heberle, T.A. Harroun, and J. Katsaras. Molecular structures of fluid phosphatidylethanolamine bilayers obtained from simulation-to-experiment comparisons and experimental scattering density profiles. *J Phys Chem B*, 119(5):1947–1956, 2015.
